# Improving the Microbiological Safety of Raw Meat Through Visible Blue–Violet Light Irradiation

**DOI:** 10.3390/foods15040690

**Published:** 2026-02-13

**Authors:** Anna Angela Barba, Gaetano Lamberti

**Affiliations:** 1 Dipartimento di Farmacia, Università degli Studi di Salerno, Via Giovanni Paolo II, 132, 84084 Fisciano, SA, Italy; aabarba@unisa.it; 2 Dipartimento di Ingegneria Industriale, Università degli Studi di Salerno, Via Giovanni Paolo II, 132, 84084 Fisciano, SA, Italy

**Keywords:** visible-light irradiation, blue–violet LED, microbiological safety, raw meat handling, post-processing contamination control, non-thermal food preservation processes

## Abstract

The interruption of primary conservation procedures during food handling and preparation represents a critical operational phase for food microbiological safety, especially in environments characterized by repeated manipulation and continuous human presence. This study investigates the application of visible blue–violet light irradiation as a non-thermal process to mitigate microbial proliferation during post-processing handling of raw meat. Raw beef hamburgers, selected as the food model substrate, were subjected to irradiation using a blue–violet LED system operating in the 405–420 nm range and compared with non-irradiated controls under ambient and refrigerated conditions representative of real handling scenarios. Microbiological dynamics were evaluated through time-resolved enumeration of total aerobic mesophilic bacteria and Enterobacteriaceae, while concurrent measurements of moisture loss, texture, and color were performed to assess process-related effects on macroscopic product quality. Visible-light irradiation significantly reduced the rate of microbial growth during handling, with irradiated samples consistently exhibiting lower microbial loads than controls, particularly under ambient conditions (e.g., twofold after 24 h). Under refrigeration, irradiation contributed to stabilizing microbial levels over time, indicating a synergistic effect with low-temperature storage. From a process perspective, irradiation induced moderate and progressive changes in physicochemical attributes, primarily associated with surface dehydration and color variation, without abrupt quality degradation. These results demonstrate that visible blue–violet light irradiation can be integrated as a continuous, non-UV intervention to enhance the microbiological safety of raw meat during post-processing handling, supporting its potential role as an environmental control strategy in food-handling systems.

## 1. Introduction

### 1.1. Microbiological Risk During Food Handling

In industrial and commercial food chains, microbiological safety is challenged not only by the intrinsic perishability of raw materials but also by the handling-intensive nature of operations such as portioning, mincing, mixing, shaping, packaging opening/re-closing, temporary storage, and repeated transfers between surfaces and utensils. Raw meat preparations (e.g., hamburgers) are a paradigmatic case: mincing and forming increase the exposed surface area, redistribute microorganisms throughout the mass, and create frequent opportunities for cross-contamination between raw products, contact surfaces, personnel, and the surrounding environment. These processes occur in settings where productivity constraints demand rapid workflows and where environmental control measures must be compatible with continuous occupancy.

From an industrial/commercial perspective, “safety” is therefore not just a matter of delivering a microbiologically compliant product at the factory gate; it is also the result of what happens after packaging is opened and during subsequent handling, including professional kitchens, retail back-of-house operations, and food-service preparation areas. In these contexts, interventions must be scalable, operator-compatible, and ideally continuous, because contamination events can occur repeatedly and unpredictably across time.

### 1.2. The “Meat Usability Window”

The concept of shelf-life traditionally refers to the period during which a product remains acceptable under specified storage conditions in its unopened state (primary shelf-life). In contrast, professional practice often hinges on a different timeframe: the period after opening and during handling, in which the product remains microbiologically acceptable for intended use. In the food literature, the closest standardized concept is secondary shelf-life (SSL), defined as the time after package opening during which the product retains a required level of quality [1,2].

In this paper, the term “meat usability window” is used to indicate, in a deliberately operational sense, the handling-time interval in which raw meat preparations can be managed (portioning, shaping, short-term holding, and preparation) without crossing microbiological acceptability limits that would reasonably raise food-safety concerns. The usability window is therefore:

• Context-dependent (temperature abuse risks, staff turnover, sanitation routines);
• Dynamic (it is affected by repeated contamination inputs);
• Actionable (it can be extended by strategies that reduce contamination pressure or slow growth).

Importantly, extending the usability window is not necessarily equivalent to claiming an extended labeled shelf-life. Rather, it can be framed as a risk-reduction measure during handling, and—if consistently effective—may represent a rational starting point toward later shelf-life extensions, provided that the regulatory and validation frameworks are addressed appropriately.

EFSA’s guidance highlights the complexity of setting time limits after opening because extrinsic conditions and contamination routes change after opening [2]. This reinforces the need for interventions designed for the opened/handling phase, particularly in professional environments.

### 1.3. Contamination Control as the Lever

**Why visible-light approaches are attractive**

Because the usability window is strongly influenced by contamination pressure, one pragmatic way to extend it is to (i) reduce the rate of re-contamination from surfaces/air and (ii) suppress microbial proliferation in the near-field environment during handling. Conventional approaches exist, but each has limitations in continuously occupied workspaces:

• Thermal methods are incompatible with raw product handling and would alter product quality;
• UV-C disinfection is effective but raises well-known concerns regarding eye/skin hazards, operational constraints, and the need to prevent unsafe exposure in occupied environments [3,4].

These constraints motivate non-thermal, non-UV strategies that can be operator-safe and potentially applied as continuous irradiation in the background of routine operations.

Within this framework, violet/blue visible light (≈380–480 nm, with a strong emphasis around ~405 nm) has emerged as a promising antimicrobial approach. Unlike UV-C, the mechanistic basis of 405 nm antimicrobial action is widely linked to photo-excitation of endogenous microbial chromophores (notably porphyrins), leading to reactive oxygen species (ROS) generation and oxidative damage [5,6,7].

Crucially for professional food-handling settings, 405 nm technology has been repeatedly discussed in the context of environmental decontamination under occupancy, particularly in healthcare environments [8,9]. This suggests a credible translational pathway to food-service environments, where the goal is not sterilization but continuous suppression of contamination during work.

The key gaps that motivate the present study can therefore be summarized as:

1. Non-UV continuous decontamination compatible with occupied food-handling spaces;
2. Operator-safe implementation that does not disrupt workflow;
3. Evidence connecting environmental irradiation to practical handling outcomes, i.e., whether contamination suppression can translate into a longer usable handling timeframe for raw meat preparations.

### 1.4. State of the Art

**Bacteriostatic/bactericidal effects of blue–violet visible light**

Seminal work has shown that 405 nm LED exposure can inactivate a broad range of bacterial pathogens. Maclean, MacGregor [5] reported inactivation of bacterial pathogens under a 405 nm LED array, providing early quantitative evidence that visible light—at sufficient dose—can be antimicrobial. Murdoch, Maclean [6] extended this evidence across multiple genera (including *Salmonella*, *Shigella*, *Escherichia*, *Listeria*, *Mycobacterium*) in liquids and on exposed surfaces, supporting the concept of environmental/near-field decontamination using 405 nm irradiation. Food-relevant pathogens have also been specifically examined: Endarko, Maclean [10] showed strong inactivation of *Listeria monocytogenes* under high-intensity blue–violet exposure, and highlighted that efficacy is concentrated in the ~400–450 nm region, consistent with endogenous photosensitizer absorption. Mechanistic studies have reinforced ROS-mediated damage models. For example, McKenzie, Maclean [7] reported loss of membrane integrity markers after 405 nm exposure, supporting oxidative injury as a key route to inactivation. Importantly, the technology has been positioned as compatible with continuous room decontamination: Maclean, MacGregor [8] reported environmental decontamination in an occupied hospital isolation room using high-intensity narrow-spectrum light centered at 405 nm. Hospital applications are still under investigation [11].

In parallel, the food-sector literature has matured from proof-of-principle to applied studies and reviews. A dedicated review in *Trends in Food Science & Technology* framed blue LEDs as a “green” non-thermal technology for microbial inactivation in foods and food environments [12]. Empirical food-relevant studies have investigated efficacy under refrigeration and/or on food-associated microorganisms. For example, Kim, Da Jeong [13] evaluated 405 nm LED effects against *Bacillus cereus*, *Listeria monocytogenes*, and *Staphylococcus aureus* at refrigerated conditions, combining inactivation kinetics with mechanistic indicators. More recently, Chen and Cheng [14] demonstrated that blue 405 nm LED light can inactivate bacterial pathogens on substrates and packaging materials relevant to food processing, strengthening the case for surface/environment applications rather than only direct product irradiation.

Across organisms and settings, the literature indicates that 405 nm visible light can range from bacteriostatic suppression to bactericidal inactivation, depending on irradiance, dose, microbial species, and the optical/physicochemical environment [9]. This dose–response nature aligns well with the present paper’s handling-oriented aim: in professional kitchens or industrial handling rooms, the objective may be continuous suppression and risk reduction, not terminal sterilization.

Recent studies support the feasibility of blue–violet antimicrobial visible light not only in controlled laboratory matrices but also across multiple food-contact substrates and packaging materials relevant to processing environments, and in real occupied settings using continuous irradiation of high-touch surfaces [13,14,15,16].

Compared with cold plasma [17], antimicrobial coatings [18] and other non-thermal treatments [19]—which typically require enclosed treatment steps, surface application/renewal, and additional validation for food-contact use—blue–violet (≈405 nm) visible light can be deployed as a continuous, non-thermal, non-chemical intervention during routine handling in occupied workspaces, providing real-time suppression of microbial accumulation without interrupting operations.

Alongside academic studies on the antimicrobial effects of blue–violet visible light, several patents have addressed the engineering implementation of this approach for environmental microbial control. These inventions typically focus on lamp architecture, wavelength selection, thermal management, and safe operation in occupied environments, rather than on introducing new biological mechanisms. For example, patent WO 2018/020527 [20] describes an LED lamp structure designed to reduce environmental microbial load through visible blue–violet irradiation, explicitly avoiding ultraviolet wavelengths and their associated health risks. The patented solution is based on the combination of multiple blue–violet LEDs emitting in narrowly defined wavelength ranges (approximately 405–420 nm), optionally combined with white LEDs to ensure usability as a general lighting source. A key feature of this approach is its compatibility with continuous operation in environments frequented by humans, overcoming limitations typical of UV-based systems. The experimental work presented in this study employs one such visible-light LED implementation, commercially referred to as *Biovitae*, (The commercial name of the light source is mentioned only for transparency and reproducibility of the experimental conditions) a commercially available LED-based visible-light source designed to emit in the blue–violet region of the spectrum (nominally within 400–420 nm, i.e., non-UV), and implemented within a broader spectral visible emission suitable for white-light illumination, which throughout the work it is treated as a “visible blue–violet LED irradiation device” and its performances are evaluated exclusively through measurable microbiological and physico-sensory endpoints.

While the antimicrobial effect of visible light may arise from the combined action of multiple spectral components, the present study does not aim to resolve the relative contribution of individual wavelength bands nor to evaluate or compare commercial technologies, but rather to investigate the effects of visible blue–violet light irradiation on microbiological safety during raw meat handling, within the framework established by the scientific literature.

### 1.5. Market Rationale

**Reducing avoidable waste by extending the usable handling timeframe**

Food waste and food safety are linked: when uncertainty arises about microbiological acceptability during handling, operators may discard food as a conservative measure, especially under time pressure. At the EU level, food waste is associated with major economic losses: the European Parliament summarizes the costs of food waste at about €132 billion in market value [21]. The European Commission reports that restaurants and food services contribute a meaningful share of EU food waste (reported as 14 kg per inhabitant in the Eurostat-based breakdown presented on the Commission’s food waste page, [22]). For the hospitality sector, WRAP reports that food waste costs the hospitality and food service sector £3.2 billion per year, corresponding to an average of ~£10 k per outlet per year [23]. WRAP’s earlier sector analysis also quantified that even small percentage reductions translate into substantial savings at scale [24]. At the global level, the economic toll of food loss and waste is commonly estimated at around USD 1 trillion annually [25,26].

In this context, a device/platform capable of extending the usable handling timeframe of raw meat preparations—by continuously reducing contamination pressure in the preparation environment—has a plausible value proposition: even a single-digit percentage reduction in avoidable waste and “precautionary discard” could generate meaningful savings for operators, in addition to the non-monetary value of improved risk management and process robustness.

### 1.6. Aim of This Work

The aim of this work is to address the following research question: Can continuous blue–violet visible-light irradiation (nominally 400–420 nm) be used as a non-thermal, operator-compatible intervention to stabilize microbial proliferation during dynamic post-processing handling of raw meat (i.e., repeated manipulation with ongoing contamination pressure)? Specifically, we evaluate whether irradiation (i) reduces the time-dependent increase in microbiological indicators during handling, thereby extending the microbiologically acceptable handling timeframe (“meat usability window”), (ii) does so without causing abrupt deterioration of macroscopic quality attributes (moisture loss, texture, and color), and (iii) provides an additive stabilizing effect when combined with refrigeration. To address these endpoints, the study combines time-resolved microbiological enumeration (TVC and Enterobacteriaceae) with parallel quality measurements under handling scenarios representative of practical conditions.

## 2. Materials and Methods

**Figure 1:**
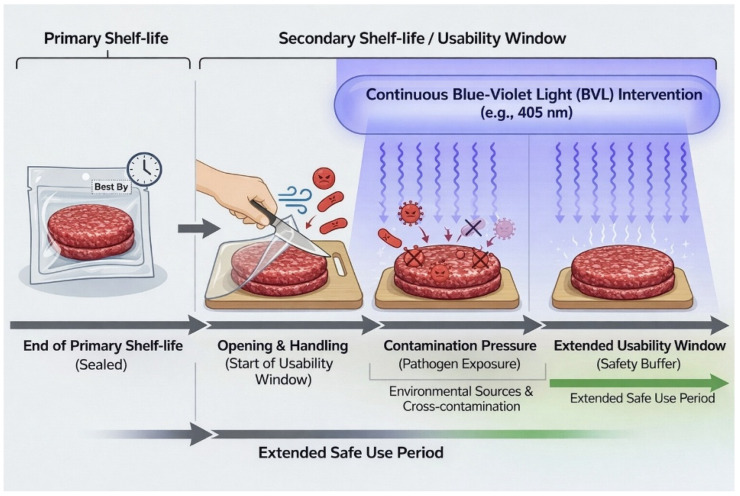
Experimental approach scheme.

The experimental approach adopted in this study is schematically summarized in Figure 1. The figure illustrates the post-processing handling phase occurring after package opening, during which raw meat is exposed to repeated manipulation and environmental contamination sources. Within this framework, visible blue–violet light irradiation is positioned as a continuous background intervention aimed at reducing contamination pressure during handling, rather than as a terminal decontamination or preservation treatment. This conceptual representation clarifies the operational meaning of “handling-time microbiological safety” addressed in the present work and guides the interpretation of the experimental design described in the following sections.

### 2.1. Visible-Light Irradiation System

**Figure 2:**
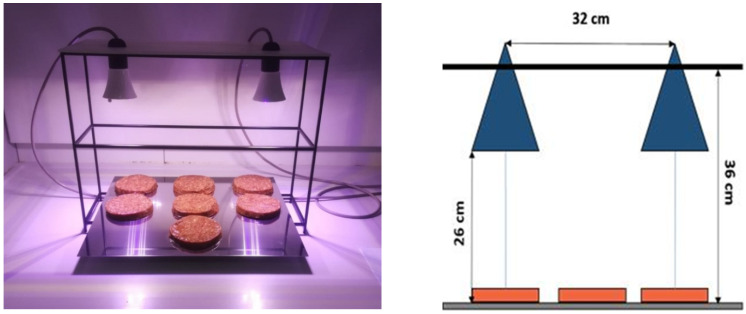

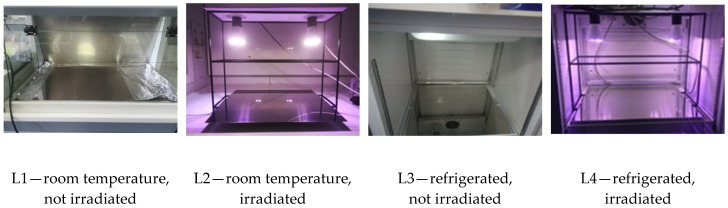
Irradiation configuration (**above**) and lines experimental set-up preparation (**below**).

The device operates as a continuous-spectrum visible-light source (approximately 400–780 nm), emitting white light for general illumination while incorporating a controlled blue–violet component used for the irradiation protocol (nominally within 400–420 nm, non-UV). The emission spectrum of the source has a dominant peak centered at approximately 410 nm (400–420 nm), as characterized by spectroradiometric measurements [27]. Based on the validated characterization reported, the irradiance at the sample surface was on the order of 1 mW cm^−2^ when samples were positioned at approximately 25 cm from the LED panel. In the present setup, the sample–light distance was maintained at ~25 cm (Figure 2). Cumulative light doses can be directly calculated as the product of irradiance and exposure time and are reported for each handling duration.

The configuration of the irradiated area is detailed in the following. Hamburger substrates have been subjected to irradiation based on defined exposure areas and controlled positioning of samples relative to the lamp(s), with paired irradiated vs. non-irradiated conditions handled in parallel.

### 2.2. Hamburger Samples and Initial Storage

Hamburger substrates (10 cm diameter, 1.5 cm thickness) were delivered under refrigerated conditions and stored at approximately 4 °C prior to the beginning of the monitoring protocol.

At the start of each monitoring line (time zero, T0), packages were opened and samples were placed on stainless-steel trays according to the assigned line (irradiated vs. control; ambient vs. refrigerated) (see Figure 2).

Spatial uniformity of irradiation across the illuminated area has been assessed for a very similar LED system by irradiance mapping at multiple positions, showing minimal variation between the center and edges of the exposed surface [27]; comparable uniform exposure conditions are therefore expected in the present study.

### 2.3. Study Lines and Handling Conditions

The experimental plan was structured into four main “study lines” intended to reproduce realistic food-handling scenarios after interruption of primary preservation (e.g., opening of packaging and intermittent manipulation during working shifts).

In the study, the four lines were:

• L1: ambient conditions, non-irradiated (control)
• L2: ambient conditions, irradiated
• L3: refrigerated conditions (~4 °C), non-irradiated (control)
• L4: refrigerated conditions (~4 °C), irradiated

For each line, samples were subjected to periodic manipulation made by humans without gloves, for a duration of 30–60 s, to mimic a “penalizing” handling scenario (hands-on manipulation) while maintaining the same handling schedule between irradiated and non-irradiated controls.

Environmental microbiological monitoring of air (CFU m^−3^) and contact surfaces (CFU cm^−2^) was not performed and should be considered in future studies to independently quantify background contamination sources during handling.

A schematic overview of the experimental setup and study lines is reported in Figure 2. The figure depicts the irradiation geometry, sample positioning, and the four experimental lines (L1–L4) combining storage temperature (ambient or refrigerated) and irradiation condition (irradiated or non-irradiated). Irradiated samples and their corresponding controls were handled in parallel under identical manipulation schedules, allowing the effects of visible blue–violet light irradiation to be evaluated independently of handling frequency and environmental exposure. Each test has a code built this way: [Line number-] + [Time] + [R if refrigerated] + [I if irradiated] + [NI if not irradiated]. For example, L3-T24hRNI means Line 3, 24 h from the beginning of the test, refrigerated, not irradiated.

### 2.4. Sampling Schedule

Monitoring was conducted over a total time window of 33 h, selected to span multiple operational time blocks compatible with professional food preparation contexts.

Sampling was performed at predefined times with higher frequency early in the monitoring (particularly for ambient-condition lines) and longer intervals for refrigerated lines, as detailed in the following.

### 2.5. Microbiological Analyses

#### 2.5.1. Total Viable Count (TVC)

The total microbial load (mesophilic aerobic microorganisms) was determined using a horizontal colony-count method aligned with ISO 4833-1:2013 (colony count at 30 °C). In brief, sample homogenates were serially diluted and plated on Plate Count Agar (PCA), followed by incubation under aerobic conditions and enumeration as CFU/g [28].

#### 2.5.2. Enterobacteriaceae Enumeration

Enterobacteriaceae were enumerated following the horizontal colony-count approach of ISO 21528-2:2017, using VRBG agar and incubation conditions consistent with the standard, with results expressed as CFU/g [29].

#### 2.5.3. Interpretation of Microbiological Quality (Contextual Thresholds)

The general EU framework on microbiological criteria for foodstuffs is defined for aerobic colony count (*m* = 5 × 10^5^ CFU/g; *M* = 5 × 10^6^ CFU/g) and for *E. coli* (*m* = 1000 CFU/g; *M* = 10,000 CFU/g) [30]. For contextual interpretation of results in a “process-hygiene/acceptability” sense, a three-class scheme is defined for minced meat (satisfactory < *m*, *m* < acceptable < *M*, unsatisfactory > *M*).

### 2.6. Physico-Sensory Measurements

#### 2.6.1. Water Loss

Free water loss was quantified gravimetrically as the percentage mass loss relative to T0 for each sample at each sampling time. The water content of each sample has been measured by a thermobalance Ohaus mod MB45 (Ohaus, Parsippany, NJ, USA), heating the sample up to 110 °C and measuring its mass (following the rule ASTM D 2216–98: Standard Test Method for Laboratory Determination of Water (Moisture) Content of Soil and Rock by Mass). Water loss percentage was calculated as the ratio between the decrease in mass and the initial sample mass.

#### 2.6.2. Texture

Texture was assessed by a controlled compression test using a Texture Analyzer TA.XT.Plus (Stable Micro System, Godalming, UK). For each sample, a probe compressed the hamburger to a fixed deformation level (50% of the sample thickness), and the maximum compression force (*F_max_*) was extracted as an operational index of compactness/firmness (higher *F_max_* → higher compactness at the same imposed deformation). The compression force was measured by a, using a cylindrical tool made in Perspex (2 cm diameter and 4 cm height). The force was registered during a compression test carried out by moving the tool down at 0.5 mm/s.

#### 2.6.3. Color and Luminosity Measurements

Surface color and luminosity were measured instrumentally using the Konica Minolta Spectrophotometer CM-700d, and expressed in the CIE L*a*b* space. Color difference over time was computed as a Euclidean distance (ΔE) in CIELAB, consistent with ISO/CIE guidance for the CIE 1976 L*a*b* color space [31]. A value of ΔE between 6 and 12 means that the difference between the two samples is significant, and a value larger than 12 means that the difference between them is very large. The a* and b* color coordinates were recorded but are not shown, as they did not provide additional independent information beyond ΔE for the applied handling context considered.

### 2.7. Statistical Analysis

Data analysis was performed on three independent experimental replicates (*n* = 3). Microbiological counts (CFU/g) were log-transformed prior to inferential statistics and are reported as log10 (CFU/g). For each thermal regime (ambient: L1 vs. L2; refrigerated: L3 vs. L4), differences between irradiated and non-irradiated samples over time were assessed by repeated-measures ANOVA, accounting for correlated measurements across sampling times within the same replicate. When a significant irradiation effect was detected, post hoc pairwise comparisons between irradiated and non-irradiated conditions at each sampling time were conducted with Bonferroni correction for multiple comparisons. Statistical significance was set at *p* < 0.05.

For all experimental conditions, microbiological and physico-sensory measurements were obtained from three independent experimental replicates conducted on separate sample sets. Reported values represent mean ± standard deviation. While the study was not designed for predictive modeling or population-wide inference, the replication level adopted is consistent with exploratory and applied food-handling studies and sufficient to support the identification of systematic trends relevant to process-level decision making.

## 3. Results

### 3.1. Microbiological Results

The effects of visible blue–violet light irradiation on microbial dynamics during post-processing handling were evaluated by monitoring TVC and Enterobacteriaceae under ambient and refrigerated conditions. The results are presented as time-resolved evolutions for irradiated and non-irradiated samples, allowing direct comparison of microbial behavior under identical handling schedules. This approach enables assessment of whether continuous visible-light irradiation can stabilize microbial loads during handling rather than acting as a terminal decontamination step.

**Figure 3:**
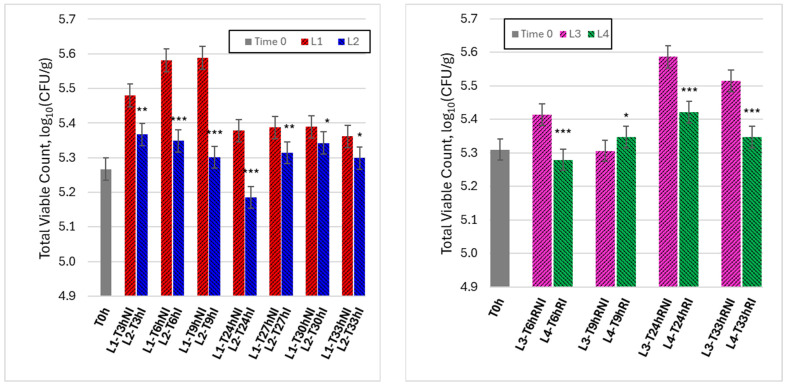
Time evolutions of TVC in Colony-Forming Units per gram (CFU/g) under different experimental conditions. **Left graph**: lines 1 and 2 (non-irradiated and irradiated samples, kept at room temperature). **Right graph**: lines 3 and 4 (non-irradiated and irradiated samples, refrigerated). Data are reported as mean ± SD (n = 3). Microbial counts were log10-transformed prior to statistical analysis. Asterisks indicate statistically significant differences between irradiated and non-irradiated samples at the same time point (* *p* < 0.05; ** *p* < 0.01; *** *p* < 0.001).

Figure 3 shows the time evolution of TVC for raw meat samples handled under ambient conditions (lines L1 and L2, left panel) and refrigerated conditions (lines L3 and L4, right panel), comparing non-irradiated controls with irradiated samples.

Under ambient conditions, non-irradiated samples (L1) exhibited a rapid increase in TVC during the early handling phase, reaching peak values within the first hours, followed by a partial decrease at longer times. In contrast, irradiated samples (L2) displayed a consistently attenuated microbial increase, with lower TVC values at all corresponding time points. The divergence between L1 and L2 became particularly evident during the period of highest microbial proliferation, indicating that visible-light irradiation effectively reduced the rate of microbial growth during ambient handling. When expressed in terms of time required to reach a comparable microbiological level, visible-light irradiation resulted in a measurable extension of the usability window. Specifically, irradiated samples reached the same TVC level observed in non-irradiated controls after approximately 6–9 h of handling with a delay of about 3–6 h, depending on the microbial indicator considered. This time shift provides an operational quantification of usability window extension under ambient handling conditions.

Under refrigerated conditions, overall microbial dynamics were slower for both lines, reflecting the inhibitory effect of low temperature. Nevertheless, a systematic difference between non-irradiated (L3) and irradiated (L4) samples was still observed. While L3 showed a progressive increase in TVC over time, irradiated samples (L4) maintained comparatively lower and more stable counts throughout the monitoring period. These results indicate that visible-light irradiation provides an additional stabilizing effect even when refrigeration is already limiting microbial growth, supporting its role as a complementary control measure during handling. Although refrigeration markedly reduced overall microbial growth, irradiated samples consistently exhibited lower log_10_ (CFU/g) values than non-irradiated refrigerated controls at several time points, indicating that visible-light irradiation provides an additional stabilizing effect beyond low-temperature storage alone.

**Figure 4:**
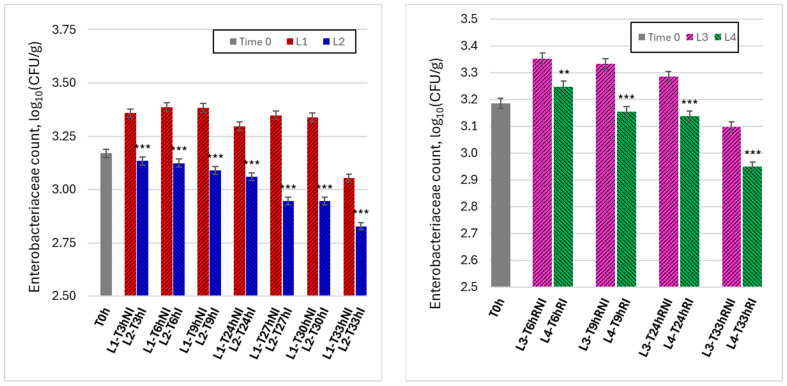
Time evolutions of Enterobacteriaceae in Colony-Forming Units per gram (CFU/g) under different experimental conditions. **Left graph**: lines 1 and 2 (non-irradiated and irradiated samples, kept at room temperature). **Right graph**: lines 3 and 4 (non-irradiated and irradiated samples, refrigerated). Data are reported as mean ± SD (n = 3). Microbial counts were log10-transformed prior to statistical analysis. Asterisks indicate statistically significant differences between irradiated and non-irradiated samples at the same time point (* *p* < 0.05; ** *p* < 0.01; *** *p* < 0.001).

Figure 4 reports the time evolution of Enterobacteriaceae counts under the same experimental conditions as Figure 3, offering a more specific indicator of hygiene-related microbial behavior during handling.

Under ambient conditions (L1 vs. L2, left panel), non-irradiated samples (L1) showed a marked increase in *Enterobacteriaceae* counts during the early handling phase, followed by sustained elevated levels over time. In contrast, irradiated samples (L2) exhibited significantly lower counts at all observation times, with a progressive reduction relative to controls as handling time increased. This pattern indicates that visible-light irradiation was particularly effective in suppressing Enterobacteriaceae proliferation during ambient handling, where contamination pressure and growth conditions are most favorable.

Under refrigerated conditions (L3 vs. L4, right panel), Enterobacteriaceae levels were generally lower and more stable than under ambient conditions. Nevertheless, irradiated samples (L4) consistently exhibited lower counts than non-irradiated controls (L3), especially after longer handling times. The persistence of this difference suggests that irradiation contributes to limiting the accumulation of hygiene-relevant microorganisms even under temperature-controlled conditions, reinforcing its potential role as a continuous background intervention rather than a one-time inactivation step. Under refrigerated conditions, the usability window extension was smaller but still detectable, with irradiated samples showing a delay of approximately 3 h in reaching comparable *Enterobacteriaceae* levels relative to non-irradiated refrigerated controls at late handling times.

### 3.2. Physico-Sensory and Handling-Related Quality Results

In parallel with microbiological monitoring, the effects of visible-light irradiation on selected physico-sensory properties relevant to raw meat handling were evaluated. Water loss, texture evolution, and color changes were monitored over time under irradiated and non-irradiated conditions, both at ambient and refrigerated temperatures, with respect to initial characteristics, summarized in Table 1. These parameters were chosen to assess potential quality trade-offs associated with irradiation during post-processing handling.

**Figure 5:**
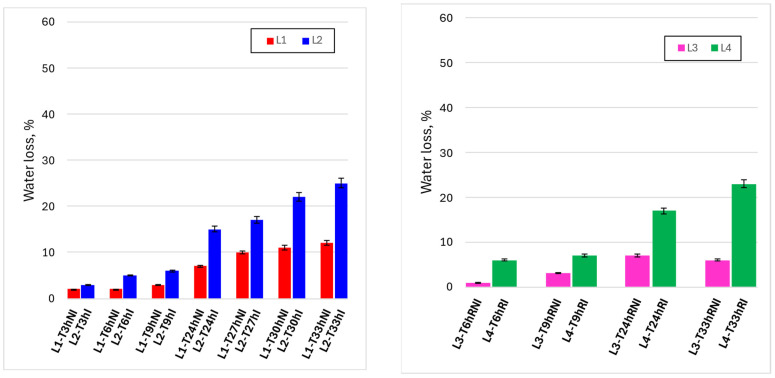

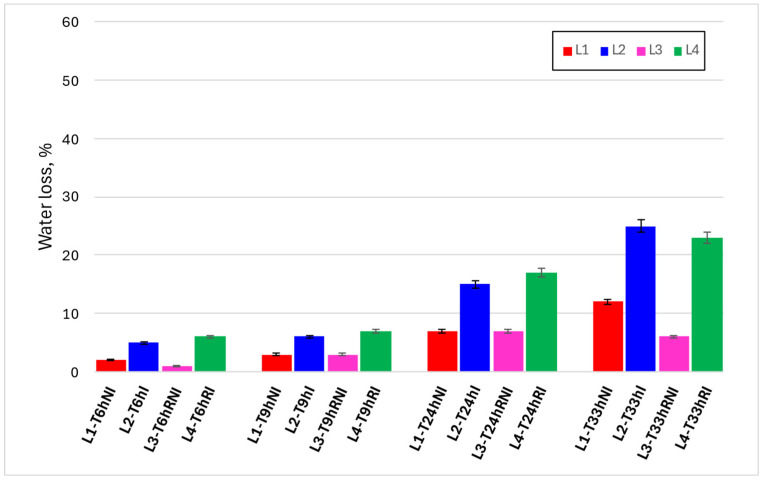
Time evolutions of water loss, %, under the different experimental conditions. **Upper left graph**: lines 1 and 2 (non-irradiated and irradiated samples, kept at room temperature). **Upper right graph**: lines 3 and 4 (non-irradiated and irradiated samples, refrigerated). **Lower graph**: comparison between the four lines, for the same observation times.

Figure 5 reports the time evolution of gravimetric water loss under the different experimental conditions, allowing a direct comparison between irradiated and non-irradiated samples under both ambient (L1–L2) and refrigerated (L3–L4) handling regimes. Under ambient conditions (upper left panel), water loss increased progressively with handling time in both lines, reflecting the combined effect of exposure to air and repeated manipulation. However, irradiated samples (L2) consistently exhibited higher water loss than non-irradiated controls (L1), with the divergence becoming more pronounced at longer times. In particular, after 24 h and beyond, the difference between L1 and L2 increased markedly, indicating that irradiation was associated with an acceleration of dehydration during prolonged ambient handling.

Under refrigerated conditions (upper right panel), overall water loss was substantially lower for both lines, confirming the dominant mitigating effect of low temperature on dehydration phenomena. Nevertheless, the same qualitative trend was observed: irradiated samples (L4) showed higher water loss than non-irradiated controls (L3) at corresponding time points, especially at extended handling times. The lower panel, where all four lines are compared at the same observation times, highlights that temperature remains the primary factor controlling water loss, while irradiation acts as a secondary but systematic contributor.

Overall, Figure 5 indicates that visible-light irradiation induces a moderate and time-dependent increase in water loss, which is strongly modulated by storage temperature and becomes relevant mainly during prolonged handling at ambient conditions.

**Figure 6:**
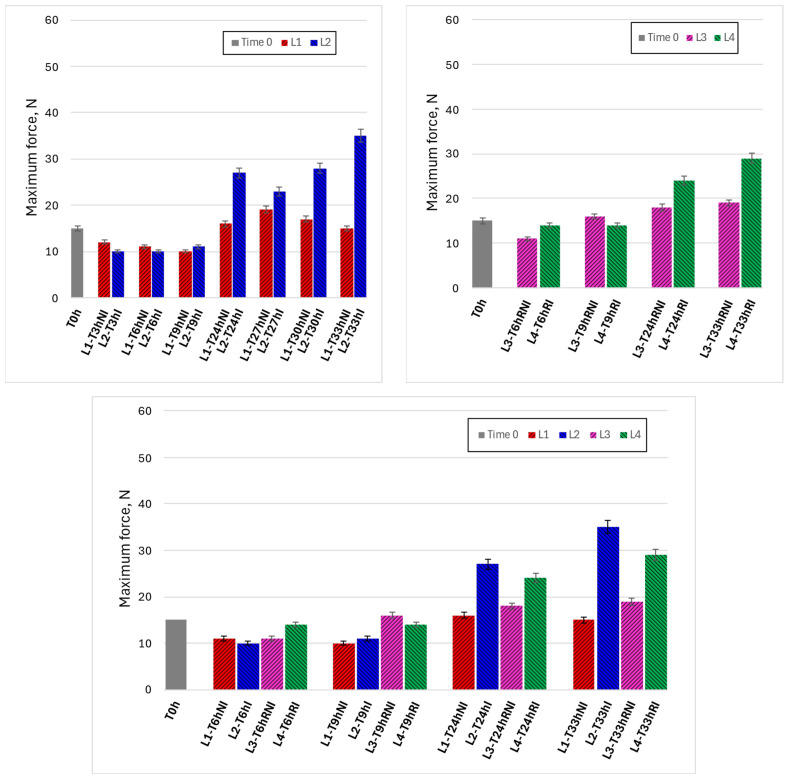
Time evolutions of maximum compression force under different experimental conditions. **Upper left graph**: lines 1 and 2 (non-irradiated and irradiated samples, kept at room temperature). **Upper right graph**: lines 3 and 4 (non-irradiated and irradiated samples, refrigerated). **Lower graph**: comparison between the four lines, for the same observation times.

Figure 6 presents the evolution of the maximum compression force (*F_max_*) as an indicator of texture and compactness during handling. Under ambient conditions (upper left panel), non-irradiated samples (L1) showed an initial decrease in *F_max_* during early handling times, followed by a gradual increase at longer times. In contrast, irradiated samples (L2) exhibited a more pronounced increase in compression force after prolonged handling, with higher Fmax values compared to controls, particularly beyond 24 h. This behavior suggests a progressive stiffening of irradiated samples during extended exposure.

Under refrigerated conditions (upper right panel), changes in compression force were generally less pronounced, and *F_max_* values remained lower than those observed under ambient handling. However, irradiated refrigerated samples (L4) still showed higher compression forces than non-irradiated controls (L3) at later time points, indicating that irradiation influenced texture evolution even when dehydration was partially mitigated by low temperature.

The comparative lower panel confirms that increases in *F_max_* correlate with the trends observed for water loss (Figure 5), suggesting a coherent and coupled behavior in which progressive dehydration leads to increased compactness. Importantly, changes in texture occurred gradually over time rather than abruptly, indicating that the effect of irradiation on mechanical properties develops progressively during handling.

**Figure 7:**
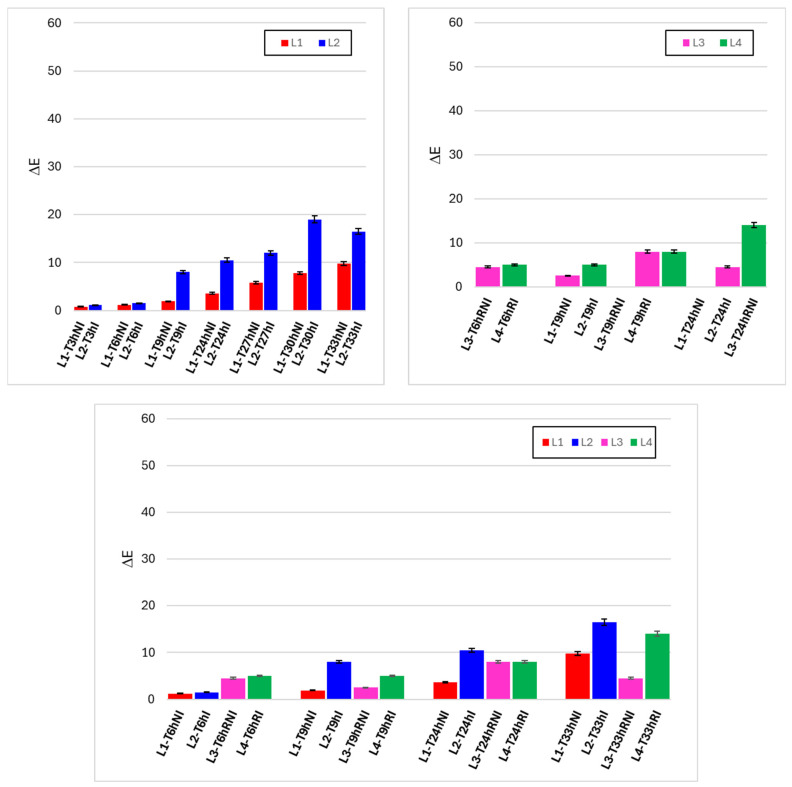
Time evolutions of ΔE under different experimental conditions. **Upper left graph**: lines 1 and 2 (non-irradiated and irradiated samples, kept at room temperature). **Upper right graph**: lines 3 and 4 (non-irradiated and irradiated samples, refrigerated). **Lower graph**: comparison between the four lines, for the same observation times.

Figure 7 illustrates the temporal evolution of color differences (Δ*E*) relative to initial conditions, providing an integrated measure of visible color change during handling. Under ambient conditions (upper left panel), Δ*E* increased with time for both irradiated (L2) and non-irradiated (L1) samples, reflecting the intrinsic sensitivity of raw meat color to handling, exposure to oxygen, and moisture loss. However, irradiated samples consistently showed higher Δ*E* values than controls at corresponding times, with the difference becoming more evident after prolonged handling.

Under refrigerated conditions (upper right panel), the increase in Δ*E* was slower and less pronounced for both lines, again confirming the protective role of low temperature. Nevertheless, irradiated samples (L4) exhibited higher Δ*E* values than non-irradiated controls (L3), particularly at the longest observation time. The lower comparative panel highlights that Δ*E* remained relatively limited during early handling but increased progressively at longer times, especially under ambient conditions with irradiation.

These results indicate that visible-light irradiation contributes to enhanced color variation over time, although the magnitude of Δ*E* remains strongly dependent on temperature and handling duration. The gradual nature of the changes suggests that color alteration develops progressively rather than as an immediate effect of irradiation.

**Figure 8:**
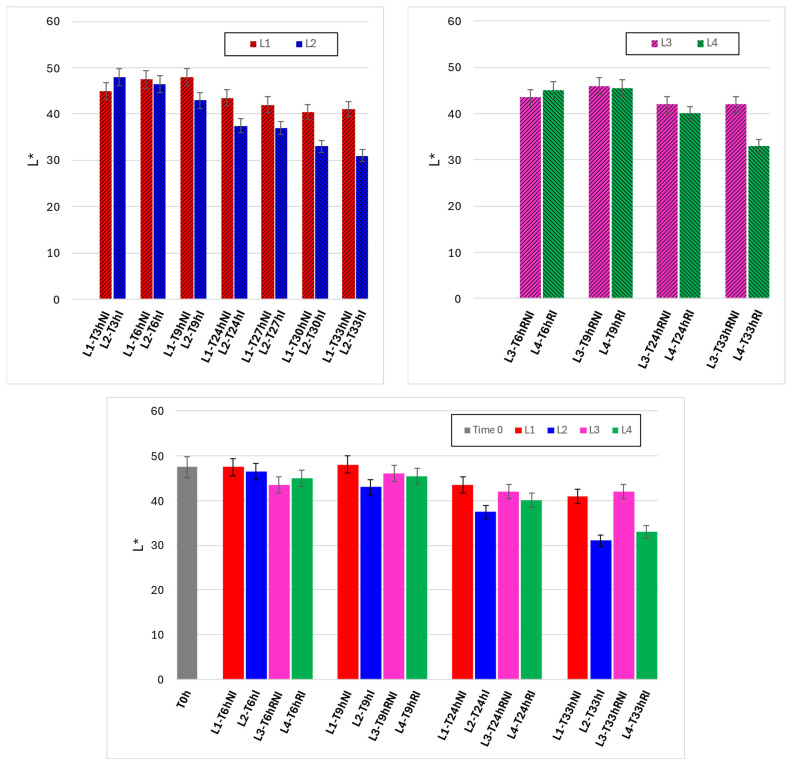
Time evolutions of L* under different experimental conditions. **Upper left graph**: lines 1 and 2 (non-irradiated and irradiated samples, kept at room temperature). **Upper right graph**: lines 3 and 4 (non-irradiated and irradiated samples, refrigerated). **Lower graph**: comparison between the four lines, for the same observation times.

Figure 8 reports the evolution of the L* coordinate, representing surface luminosity, under the different experimental conditions. Under ambient handling (upper left panel), L* values decreased over time for both irradiated and non-irradiated samples, indicating progressive darkening during exposure and manipulation. Irradiated samples (L2) generally exhibited a stronger decrease in L* compared to controls (L1), particularly at longer times, suggesting enhanced surface darkening associated with irradiation during extended handling.

Under refrigerated conditions (upper right panel), the reduction in L* was more limited, and luminosity remained relatively stable during early handling. Nonetheless, irradiated samples (L4) again showed a greater decrease in L* compared to non-irradiated samples (L3) at later time points. The lower panel comparison across all lines emphasizes that refrigeration substantially attenuates luminosity loss, while irradiation introduces a secondary, consistent contribution to surface darkening.

Taken together, Figure 7 and Figure 8 show that color changes associated with irradiation are primarily driven by progressive darkening and overall color displacement, developing gradually with handling time and remaining strongly dependent on storage temperature.

### 3.3. Integrated Interpretation

**Microbiological benefit vs. quality trade-offs**

The combined analysis of microbiological and physico-sensory results provides a coherent framework to evaluate the practical implications of visible blue–violet light irradiation during raw meat handling. From a microbiological standpoint, irradiation consistently reduced microbial proliferation rates under both ambient and refrigerated conditions, with the most pronounced benefits observed during ambient handling. This behavior aligns with the concept of bacteriostatic stabilization rather than terminal inactivation, supporting the intended use of visible-light irradiation as a continuous, operator-compatible intervention during handling phases.

These microbiological benefits must be considered alongside the observed quality-related effects. Irradiated samples exhibited moderately higher water loss over time, accompanied by gradual increases in firmness and progressive color changes, particularly under ambient conditions. Importantly, these effects developed progressively with handling duration and were strongly mitigated under refrigeration, indicating that they are not abrupt or irreversible consequences of irradiation but rather time- and condition-dependent trade-offs.

When interpreted together, the results suggest that visible-light irradiation shifts the balance between microbiological risk and quality preservation in a predictable manner. The microbiological stabilization achieved during handling may justify a controlled increase in dehydration- and color-related changes, especially in scenarios where food safety margins are critical. Moreover, the strong influence of temperature on all quality parameters highlights the potential for engineering optimization—through exposure time, irradiation geometry, and integration with refrigeration—to maximize microbiological benefits while minimizing quality impacts.

Overall, the integrated dataset indicates that visible blue–violet light irradiation can meaningfully enhance handling-phase microbiological safety, provided that its application is tuned to operational conditions. Rather than representing a simple trade-off between safety and quality, the results define an adjustable operational window in which continuous irradiation can be used to improve process robustness during raw meat handling.

## 4. Discussion

This study investigated visible blue–violet light irradiation as a handling-phase intervention for raw meat products, aiming to stabilize microbiological conditions during post-processing handling rather than claiming sterilization or classical shelf-life extension. The discussion is organized into (i) microbiological outcomes and their regulatory/benchmark context and (ii) physical/quality outcomes and their practical implications, followed by limitations and operational considerations.

### 4.1. Microbiological Outcomes

**Bacteriostatic stabilization during handling**

#### 4.1.1. Effect Magnitude and Interpretation

Across study lines, the key microbiological result is the systematic stabilization of microbial indicators under visible blue–violet irradiation, particularly evident under ambient handling conditions (L2 vs. L1), and still observable as an added margin under refrigeration (L4 vs. L3). This pattern is consistent with the established understanding of violet/blue antimicrobial light (aBL/VBL) mechanisms, where inactivation is largely attributed to endogenous photosensitizers (e.g., porphyrins, flavins) leading to oxidative stress and loss of cellular functionality, without the need for exogenous sensitizers [5].

In the current results, the most relevant framing is bacteriostatic control: irradiation appears to reduce the effective growth rate and/or contamination pressure during repeated handling exposure, rather than producing an immediate “kill step.” This aligns well with prior evidence that 405 nm technologies have lower instantaneous germicidal power than UV, but their practical advantage is the feasibility of continuous use in occupied environments, where cumulative dose and persistent action can become meaningful [32].

It should be emphasized that the present study does not directly quantify intracellular reactive oxygen species, porphyrin excitation, or specific molecular damage pathways. The mechanistic interpretation proposed here is therefore based on established evidence from the literature on antimicrobial blue–violet light, rather than on direct mechanistic measurements performed within this work. In this context, references to oxidative stress, endogenous photosensitizers, and membrane damage should be interpreted as a coherent explanatory framework consistent with prior studies, rather than as experimentally demonstrated mechanisms under the specific conditions tested here. Therefore, any reference to ROS-mediated oxidative damage and endogenous photosensitizers is intended only as a literature-supported interpretation of antimicrobial blue–violet light and should not be construed as an experimentally proven mechanism under the specific conditions tested here. This distinction is particularly relevant in handling-oriented applications, where the practical objective is the stabilization of microbial dynamics over time rather than the detailed elucidation of cellular damage pathways.

Refrigeration represents the primary control measure for limiting microbial proliferation in raw meat; however, the present results demonstrate that continuous visible-light irradiation offers an additive contribution rather than a redundant effect. Under refrigerated handling, statistically significant differences between irradiated and non-irradiated samples emerged at later time points, suggesting that irradiation primarily delays microbial accumulation when refrigeration alone becomes less effective during prolonged handling.

#### 4.1.2. Comparison with Literature

The wavelength range adopted here (blue–violet) is also coherent with comparative data showing that inactivation efficacy depends strongly on spectral peak and microbial physiology. Food microbiology studies have reported antimicrobial activity across 395–425 nm, with evidence that both ROS-dependent and photophysical pathways can contribute to membrane disruption [33]. Mechanistic work specifically at 405 ± 5 nm under refrigeration supports membrane integrity loss as a key damage pathway [7] and reports log reductions for Gram-positive pathogens at sufficiently high doses [34].

From an application perspective, the present handling-focused outcomes are also consistent with “real-surface” demonstrations in food-related environments: 405 nm LEDs have been shown to reduce pathogens on stainless steel and polymeric surfaces typical of food processing and food-service contexts [14]. This matters because, in professional handling, the contamination pressure is often driven by surface and near-field environmental reservoirs, not only by the initial load of the product.

#### 4.1.3. Regulatory/Benchmark Context for Acceptability

A useful benchmark is provided by EU microbiological process hygiene criteria (see Section 2.5.3). While the present work monitored total aerobic counts and Enterobacteriaceae during post-packaging handling, rather than verifying process hygiene at manufacturing release, the regulation still provides an interpretable reference framework: maintaining microbiological indicators in “satisfactory/acceptable” ranges during handling is consistent with the operational goal of reducing the probability that the product exceeds indicative contamination thresholds or triggers corrective actions in practice.

Overall, the microbiological results support the proposition that visible blue–violet irradiation can function as a continuous, operator-compatible barrier in professional handling scenarios, complementing (not replacing) temperature control and hygienic practices.

### 4.2. Physical/Quality Outcomes

**Dehydration-driven trade-offs (water loss, texture, color)**

#### 4.2.1. Water Loss and Texture: A Coupled Effect

The main quality-side signal is the moderate increase in water loss in irradiated lines, accompanied by the increasing maximum compression force (firmness/compactness) over time. Importantly, the direction- and time-dependence of both variables suggest a coupled mechanism dominated by surface dehydration during prolonged exposure, rather than a thermal denaturation effect (consistent with a non-thermal LED source). Refrigeration clearly mitigates both water loss and firmness increase, indicating that operational control (temperature + exposure time) can manage the trade-off.

This is consistent with the broader food-tech literature describing blue LED strategies as “non-thermal” microbial control methods, but also noting that quality attributes can be affected by oxidation/dehydration pathways depending on matrix, dose, and exposure conditions [12].

#### 4.2.2. Color Changes: Practical Significance for Handling

Color changes (ΔE and L*) also progressed with time, with irradiated samples showing somewhat larger deviations, again more pronounced at ambient temperature. In raw meat handling, color is a crucial decision cue; therefore, even modest shifts may influence usability decisions. The key practical point is that the observed changes appear gradual and likely controllable through exposure duration, geometry, and refrigeration, which supports the feasibility of engineering-based optimization (e.g., positioning, shielding, intermittent duty cycles) rather than treating color drift as a fatal limitation.

It is also worth noting that related light-based antimicrobial approaches in foods (including photodynamic strategies using sensitizers) often report the same need to balance antimicrobial efficacy and quality endpoints, reinforcing the importance of dose management and matrix-specific optimization [35].

### 4.3. Practical Implications, Limitations, and Next Optimization Steps

A central strength of the present dataset is that it reflects handling-time dynamics (ambient vs. refrigerated; irradiated vs. control), which is closer to real professional workflows than short, terminal treatments. The results suggest two actionable implications:

• Where it helps most: ambient handling scenarios, where irradiation can provide the greatest microbiological stabilization relative to controls—consistent with the concept that continuous irradiation accumulates benefit over time [32].
• What to manage: the main trade-off is dehydration-related quality drift (water loss → firmness → color), indicating that optimization should focus on dose distribution (geometry/distance), exposure scheduling, and refrigeration coupling.

Limitations that should be explicitly recognized in the paper include: (i) the dependence of efficacy on dose and surface exposure uniformity (a known constraint of aBL/VBL systems; [9] and (ii) the need to verify performance under broader operational variability (different meat batches, fat content, handling intensities, and real kitchen airflow/occupancy patterns). The generalizability of the present findings should be interpreted within the context of the experimental system adopted. Raw meat preparations are inherently heterogeneous matrices, and factors such as fat content, surface roughness, moisture distribution, initial microbial composition, and handling intensity are known to influence both microbial dynamics and light–matter interactions. Accordingly, while the observed trends—namely bacteriostatic stabilization under irradiation and progressive, dehydration-driven quality changes—are expected to be qualitatively transferable to comparable handling scenarios, quantitative outcomes (e.g., magnitude of microbial suppression or rate of quality drift) may vary across products and operational settings. This underscores the need for system-specific calibration of exposure time, geometry, and integration with temperature control when translating visible-light irradiation into different food-service or processing environments. These are natural next steps toward an engineering deployment rather than issues that undermine the proof-of-concept.

## 5. Conclusions

This study demonstrates that continuous blue–violet visible-light irradiation (405–420 nm) can act as a non-thermal, operator-compatible intervention to stabilize microbial proliferation during post-processing handling of raw meat under realistic working conditions. Rather than producing terminal decontamination, irradiation consistently delayed microbial accumulation, resulting in a measurable extension of the microbiologically acceptable handling timeframe (“meat usability window”).

Under ambient conditions, irradiated samples reached comparable TVC and Enterobacteriaceae levels several hours later than non-irradiated controls, corresponding to a usability window extension on the order of 3–6 h depending on the microbial indicator. Under refrigeration, microbial growth was already strongly constrained; nevertheless, visible-light irradiation provided an additional stabilizing effect at intermediate and late handling times, confirming an additive interaction with low temperature rather than a replacement of refrigeration.

Quality-related changes (moisture loss, texture, and color) remained moderate over the investigated handling period and did not indicate abrupt degradation associated with irradiation, supporting the compatibility of continuous visible-light exposure with practical handling requirements. Mechanistically, the observed effects are consistent with literature-reported antimicrobial blue–violet light pathways involving endogenous photosensitizers and oxidative stress, although no direct mechanistic measurements were performed in this study.

Overall, these results support the use of blue–violet visible-light irradiation as a complementary, continuous control measure for reducing microbiological risk during dynamic meat handling, addressing a critical gap between static laboratory studies and real-world food-processing operations. Future work should focus on broader multi-matrix validation and integration with environmental monitoring to further support industrial implementation.

## Figures and Tables

**Table 1:** Initial (time T0) characteristics of the hamburgers.

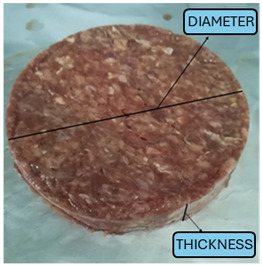	Diameter [cm]		10.9 ± 0.23
	Thickness [cm]		1.55 ± 0.12
	Weight [g]		151.5 ± 1.51
	Water [%]		46.5 ± 3.55
	Firmness	*F_max_* [N]	15.5 ± 0.59
	Color	*L*[-]	47 ± 2.56
		*a* [-]	13.7 ± 1.66
		*b* [-]	16.7 ± 1.38

## Data Availability

Data are available from the corresponding author upon reasonable request.
